# 3D-printed visualization of a double right coronary artery with intra-atrial course

**DOI:** 10.1007/s10554-021-02451-5

**Published:** 2021-10-29

**Authors:** Simon M. Frey, Philipp Brantner, Julian Gehweiler, Antonio Madaffari, Michael J. Zellweger, Philip Haaf

**Affiliations:** 1 University Hospital Basel, Basel, Switzerland; 2 University Hospital Basel, Basel, Switzerland; 3 University Hospital Bern, Bern, Switzerland

A 55-year-old male patient with atypical angina, dyspnoea, elevated cardiovascular risk (hypertension, hypercholesteremia, smoker, obesity) and a non-conclusive stress echocardiography was referred for computed tomography coronary angiography (CTCA). There was moderate coronary calcification (Calcium Score 8 (Agatston), 50. percentile) without obstructive coronary artery disease (all segments < 50%). However, CTCA showed various coronary anomalies with potential clinical impact (Fig. 1, video 1–5): the right coronary artery (RCA) originated from the right coronary cusp and early trifurcated in a right ventricular branch and an anterior and posterior double RCA, both running in the right atrioventricular groove. The posterior mid RCA then penetrated the right atrial (RA) wall at the ostium of the right atrial appendage and exhibited an intracavitary course of 40 mm (Panel 1). After the exit from RA, both RCAs re-united in anastomosis. Additionally, the apical left anterior descending coronary artery (LAD) had an 8 mm intracavitary course within the apical right ventricle. Correlation of these incidental findings with the atypical chest discomfort was deemed as unlikely, especially because there were no significant coronary stenoses. Therefore, a conservative approach with optimal secondary prevention was recommended. To the best of our knowledge, this is the first description of this combined coronary artery anomaly with potential clinical implications.

**Fig. 1:**
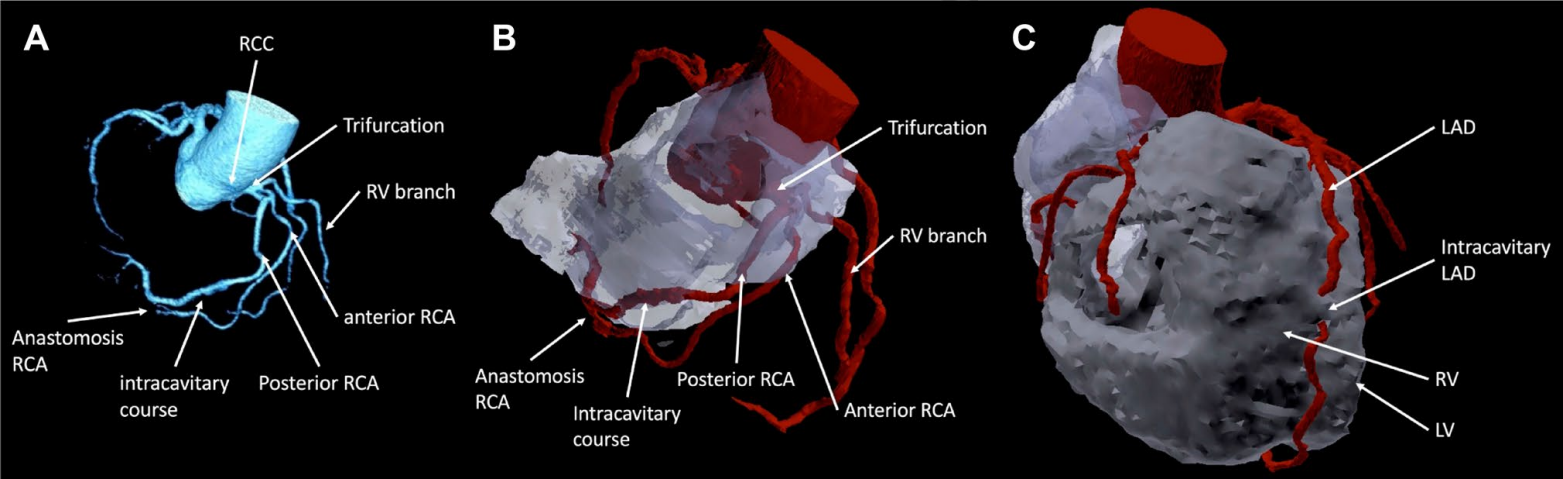
Multiple coronary anomalies. Panel **A** Multiplanar CTCA reconstruction of coronary tree showing four anomalies of the RCA (trifurcation, double RCA, intracavitary course, anastomosis). Panel **B** 3D-PDF model rendered from original CT data indicating the above mentioned RCA anomalies. The grey, transparent area represents the right atrium. Panel **C** 3D-PDF showing short intracavitary course of the mid LAD within the right ventricle. *CTCA* Computed tomography coronary angiography, *LAD* left anterior descending coronary artery, *LV* left ventricle, *RCA* right coronary artery, *RCC* right coronary cusp, *RV* right ventricle

## Discussion

Double RCA and intracavitary coronary course are extremely rare anomalies. Prevalence of intracavitary coronary course was initially reported to be very low at 0.1% [1], but contemporary studies point towards higher numbers (1.3% [2] to 1.8% [3]). Initial prevalence was presumably underestimated as detection during bypass surgery or using 2D invasive coronary angiography is difficult. Given the increasing use of advanced cardiac imaging such as CTCA, its true prevalence is likely to increase even further.

Although they are usually clinically benign, these anatomic variants may impose myriad of clinical challenges around invasive cardiac procedures, in particular if unrecognized prior to the procedure: (1) in the setting of interventional or cardiovascular surgical revascularization leading to difficulties in vessel localization as well as bypass grafting; (2) right heart catheterization leading to potential injury of the vessel; and (3) in case of electrophysiological procedures such as catheter ablation or lead device implantation. Lead device implantation at the right atrial wall or right ventricular apex in these patients could directly damage coronary arteries with intracavitary course and lead to inadvertent disruption of the vessel.

At the current time, AHA and ESC guidelines do not cover specific recommendations for such patients. Therefore, management should be tailored to the individual patient.

**Fig. 2:**
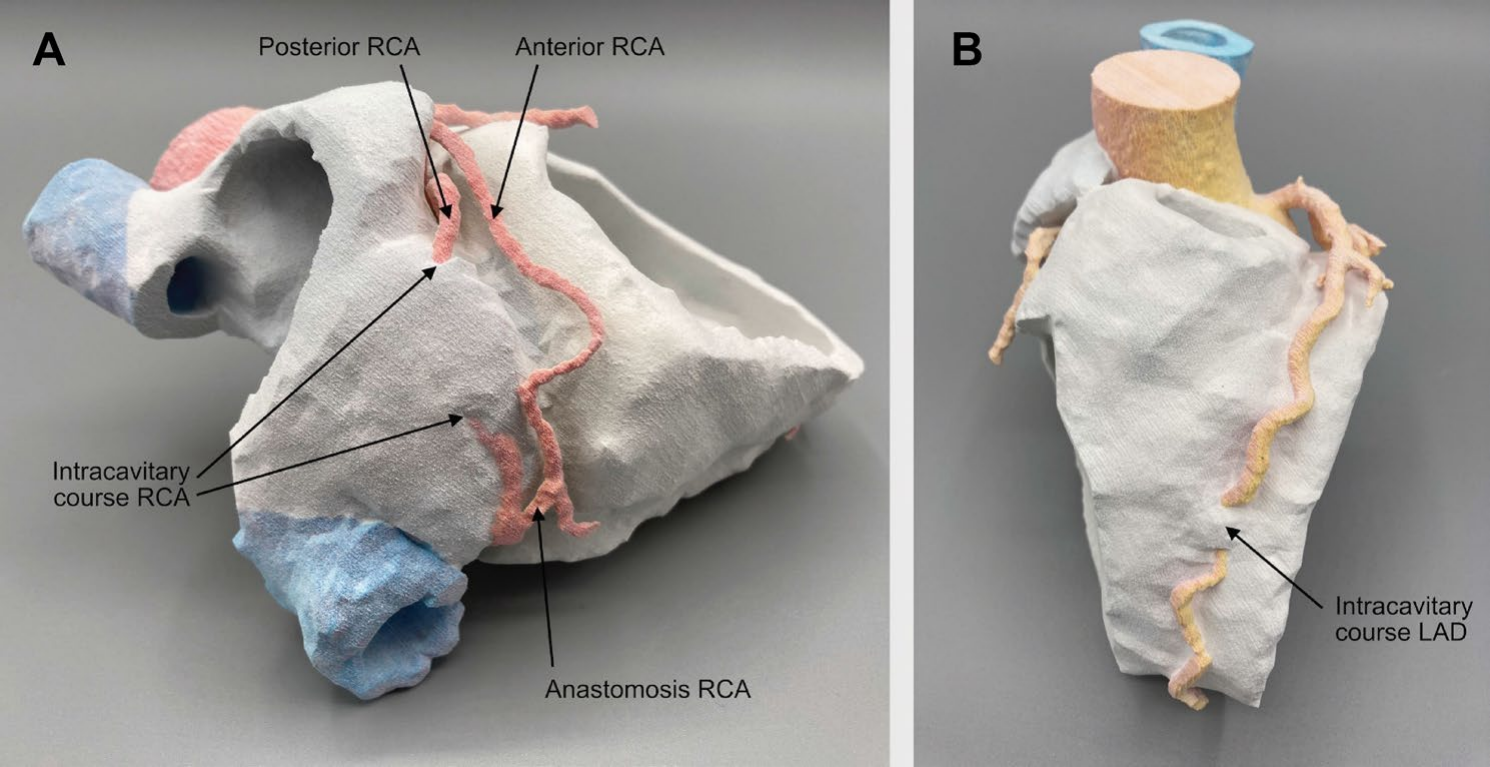
Multicolor 3-dimensional printing model. The 3D printed model was segmented in Materialise mimics and 3-matic (Materialise NV, Leuven, Belgium) and manufactured with a commercial high-resolution multijetting material printer (ProJet 660 Pro, 3D Systems, USA). Panel **A** demonstrates the intracavitary course of the posterior RCA as well as the distal anastomosis of the anterior and posterior RCA. Panel **B** shows the intracavitary course of the LAD. *LAD* left anterior descending coronary artery, *RCA* right coronary artery

3D printing is an excellent tool to demonstrate such complex anomalies to the patients affected and to colleagues who are not familiar with advanced cardiac imaging (Fig. 2, video 3). Knowledge of such cases can increase the awareness for coronary anomalies which can be a potential harm for patients during invasive cardiac procedures.

## Supplementary Information

Below is the link to the electronic supplementary material.

Supplementary material 1 (PDF 1418.5 kb)

Supplementary material 2 (MP4 4435.9 kb)

Supplementary material 3 (MP4 13389.3 kb)

Supplementary material 4 (MP4 21120.0 kb)

Supplementary material 5 (MP4 17897.8 kb)

Supplementary material 6 (MP4 10091.2 kb)

## Abbreviations

AHA: American Heart Association
CTCA: Computed tomography coronary angiography
ESC: European Society of Cardiology
LAD: Left anterior descending artery
RA: Right atrium
RCA: Right coronary artery
